# Advanced Effect of* Moringa oleifera* Bioconversion by* Rhizopus oligosporus* on the Treatment of Atopic Dermatitis: Preliminary Study

**DOI:** 10.1155/2018/7827565

**Published:** 2018-01-17

**Authors:** Sang-sun Hur, Suk-won Choi, Dong-ryul Lee, Jong-hwan Park, Tae-ho Chung

**Affiliations:** ^1^Division of Integrated Biotechnology, Joongbu University, Chungnam 32713, Republic of Korea; ^2^Factorial Research Center, Inc., Seoul 06103, Republic of Korea; ^3^Laboratory of Animal Medicine, College of Veterinary Medicine, Chonnam National University, Gwangju 61186, Republic of Korea

## Abstract

This study was conducted to determine if topical application of* Moringa oleifera* extracts and its bioconversion product fermented by* Rhizopus oligosporus* has therapeutic properties enhancement for treatment of atopic dermatitis.* Rhizopus oligosporus* (KCCM 11232P) was used to ferment* Moringa* leaves' extracts in this study. Comparison of organic acids and flavonols in* Moringa* simple extracts and their fermented product by HPLC analysis revealed that concentration of organic acids and flavonols of bioconversion product was lower than that of hot water extracts. The fermentation process is used as a nutrient for isolation of each component by microorganisms and growth of microorganisms. The results demonstrated that MF extracts effectively reduced clinical features based on macrography, scratching count, and severity scores, as well as model's serum IgE level, including histopathological analyses.

## 1. Introduction

Atopic dermatitis (AD) is a chronic inflammatory skin disease associated with cutaneous hyperreactivity to environmental triggers innocuous to normal nonatopic individuals [1]. AD is characterized by a typical sequence of progression of clinical signs such as erythema, edema/papulation, oozing/crusts, excoriations, and lichenification to atopic march [2]. AD is a systemic skin disease for which there are few satisfactory systemic therapies without involving glucocorticoids [3]. Several previous studies' results suggest that many kinds of herbal medicines may be applicable for treatment of AD without adverse effects [4].

*Moringa oleifera (M. oleifera)* is widely cultivated in tropical and subtropical regions and has been used as a vegetable and in traditional medicine [5].* Moringa oleifera* Lam. belongs to the Moringaceae family and is widely cultivated in tropical and subtropical regions. The plant has been used traditionally for treatment of skin diseases, anemia, cholera, and other illnesses, including AD [6]. In addition, many scientific studies have reported antimicrobial, anti-inflammatory, antidiabetic, and anticancer properties of* M. oleifera* [7, 8]. The extracts from mature and tender leaves of* M. oleifera* revealed potent antioxidant activity [9].

There has been increased interest in use of traditional herbal medicine to develop new therapeutic agents without steroids for AD treatment [6]. Additionally, recent double-blind, placebo-controlled, crossover studies reveal considerably effective benefits in managing clinical signs of AD by herbal plant extract bioconversion technology [10]. However, it is crucial to investigate active principles of herbal medicines for quality control and to determine their real therapeutic value in modern pharmacology [11]. Recent studies have suggested that fermentation of herbal extracts may affect therapeutic potential due to increased absorptive effect and effective molecule changing, especially by* Rhizopus* spp. bioconversion system [12].

In this study, we examined anti-AD effects* of M. oleifera* extracts (ME) and enhanced therapeutic potential of* M. oleifera* bioconversion product (MF) by* Rhizopus oligosporus (R. oligosporus)* in* in vivo* AD models. Anti-AD effects of* M. oleifera* leaves have been reported, but there have not been reports of enhancement of effect by fermentation process. We hope this preliminary report will trigger investigation specificity of* M. oleifera* bioconversion by* R. oligosporus* and its mechanism study. This is the first study where* M. oleifera* bioconversion by* R. oligosporus* could maximize therapeutic effect by reconstituting active substances.

## 2. Materials and Methods

### 2.1. Preparation and Fermentation of* M. oleifera*

Fresh leaves of* M. oleifera* were sorted to eliminate impurities and then washed with tap water. Thereafter, these leaves were washed with distilled water and drained on plastic trays. Leaves were then dried at 30°C ± 2°C for 48 hours in a ventilated hot air dryer (LTCO42, Korea). Dried leaves were crushed in a hammer mill (PC-10-F, Korea) through a sieve mesh of 10 *μ*m. Moringa extracts were used as ME.* Rhizopus oligosporus* (KCCM 11232P) was provided by the Microbiology Laboratory of KRIBB, Korea. This strain was propagated in MRS Broth (pH 5.5) at 37°C for 16 hours. Culture obtained was centrifuged at 6500 rpm for 20 minutes at 4°C and the bottom used as starter.

For preparation of MF, approximately 12.5 g of leaves powder was introduced into rice-soybean extract (350 grams of rice and soybean mixture was added to 3 liters of distilled water) of 3000 mL and then sterilized at 121°C for 20 minutes. After cooling, the mixture was inoculated with* Rhizopus oligosporus* (KCCM 11232P). Fermentation was conducted at 29°C for 120 hours. After fermentation, samples were filtered using 0.45 *μ*m pore size filters, followed by evaporation.

The total phenolic content was assessed following the modified Folin-Ciocalteu colorimetric assay procedure described by Dorman et al. [13]. Each of extracts (0.5 mL) was mixed with 0.5 mL of FC reagent and the solution was stirred vigorously by vortex and left to stand for 5 min. Finally, 2 mL of 20% Na_2_CO_3_ was added, stirred vigorously for the last time, and left to stand at room temperature for 1 hr. The mixture was then incubated further at 22°C for 90 min and the absorbance was measured at 640 nm. The total phenolic content (mg/mL) was calculated using tannic acid as standard.

For analysis of organic acids in the* Moringa* fermented extracts, 11 types of standard materials (citric, tartaric, malic, quinic, succinic, lactic, formic, acetic, propionic, gallic, and butyric) were purchased from Sigma-Aldrich Chemical Co. (St Louis, MO, USA). Samples were analyzed using HPLC (Shimadzu Co., Prominence, Japan) at 210 nm. Each sample (20 *μ*L) was injected into the HPLC system. H_2_SO_4_ (50 mM dissolved in water) solution was used as a mobile phase after adjusting pH at 2.8. Flow rate of mobile phase was maintained at 0.5 mL/minute. Column for HPLC analysis was Hi-Plex H (7.7 mm × 300 mm I.D., Agilent Technologies Co.). The method described above was detailed in Table 1. For flavonols analysis, 100 mL of each sample was centrifuged at 300 ×g for 20 minutes using ultrasonicator (3210R-DTH, Branson Ultrasonic Co., USA). The supernatant was filtered twice with Whatman filter paper (number 41), filtered with a syringe filter (0.22 *μ*m, National Scientific, USA), and 20 *μ*L of the filtrate was injected into HPLC to analyze flavonols. Flavonols of each sample were analyzed by gradient solvent system using HPLC (Waters 600, Waters Co., USA). The column was Pursuit XPs 3 C18, the column temperature was 40°C, and detector was UV detector (280 nm). For the mobile phase, distilled water was used for the A solution and acetonitrile for the B solution. Flow rate was maintained at 1 mL/minute and ratio of the solution after injection was described in Table 2. Isolated flavonols content was calculated from standard curve of the peak area for concentration of gallic acid, chlorogenic acid, vanillic acid, caffeic acid, p-coumaric acid, ferulic acid, diagenin, quercetin, apigenin, and kaempferol, respectively.

### 2.2. Animals and AD Induction

The Institutional Animal Care and Utilization Committee of Joongbu University approved all animal procedures. Male BALB/c mice (weighing 25 g) were purchased from Joong-Ang Experimental Animals Co. (Seoul, Korea) and placed in cages at a temperature between 20 and 23°C with a 12-hour light/dark cycle and a relative humidity of 50%. Animals were given commercial mouse chow (Purina, Korea) and water ad libitum.

Controlled atopic dermatitis was induced by topically applying 0.1% DNCB dissolved in acetone/olive oil (1 : 3) to the back of mice 3 times per week (Monday, Wednesday, and Friday) for 10 weeks. Mice were then housed for 3 days without further treatment.

For AD induction, mice were divided into 6 groups as AD only group (NC, negative control), topical application of* M. oleifera* extract by 5 mg/kg (T1), topical application of* M. oleifera* extract by 10 mg/kg (T2), topical application of* M. oleifera* bioconversion product by 5 mg/kg (T3), topical application of* M. oleifera* bioconversion product by 10 mg/kg (T4), and topical application of dexamethasone cream by 1 mg/kg (PC, positive control).

### 2.3. Clinical Skin Severity Score

Efficacy of* M. oleifera* extract and* M. oleifera* bioconversion product in the NC/Nga mice was evaluated by changes of severity of skin lesions (a modified SCORAD) as follows. Severity of AD-like dorsal skin lesions was assessed once a day as follows: dorsal lesions were evaluated for 5 symptoms: erythema/darkening, edema/papulation, excoriations, lichenification/prurigo, and dryness. Each symptom was graded from 0 to 3 (none, 0; mild, 1; moderate, 2; severe, 3). Clinical skin score was defined as the sum of the individual scores, ranging from 0 to 15. The dorsal skin of each mouse was photographed before, during, and after treatment.

### 2.4. Scratching Behavior

The scratching behavior was recorded on video once a day for 28 consecutive days. Specifically, the number of times a mouse scratched the dorsal skin lesion within a period of 15 minutes was calculated. Because the average number of scratches in each mouse varied daily, scratching behavior was estimated as percentage of the control calculated from mean value of the no-treatment group.

### 2.5. Detection of Serologic IgE Concentration

Blood was collected from the retroorbital area and abdominal vena cava. Levels of eosinophils and immunoglobulin E (IgE) in the blood were measured. Blood was collected from mice and transferred into EDTA-treated tubes, after which plasma was separated by centrifugation at 3,000*g* for 10 minutes at 4°C and stored at −80°C. Plasma levels of IgE were measured with ELISA kits (R&D Systems, Boston, MA, USA) according to manufacturer's instruction.

### 2.6. Histopathological Evaluation

Dorsal skins with AD-like lesions were obtained and fixed in 10% neutral formalin for histopathological observation. Tissues were routinely processed with alcohol and xylene series and embedded in paraffin. 3 *μ*m sections were prepared, stained with hematoxylin-eosin (HE), and examined by microscopy.

### 2.7. Statistical Analysis

Experimental values are given as means ± SEM. Statistical difference was determined by a two-sided Mann–Whitney *U* test. *p* < 0.05 was considered statistically significant.

## 3. Results

### 3.1. Composition of Organic Acids, Flavonols, and Total Phenolic Content in ME and MF

**Figure 1:**
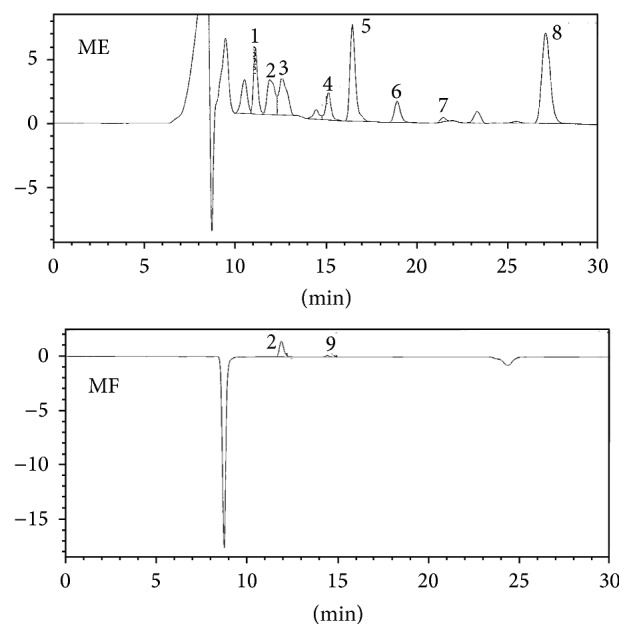
The HPLC chromatogram of ME and MF. 1, citric; 2, tartaric; 3, malic; 4, succinic; 5, lactic; 6, acetic; 7, propionic; 8, butyric; and 9, quinic.

**Figure 2:**
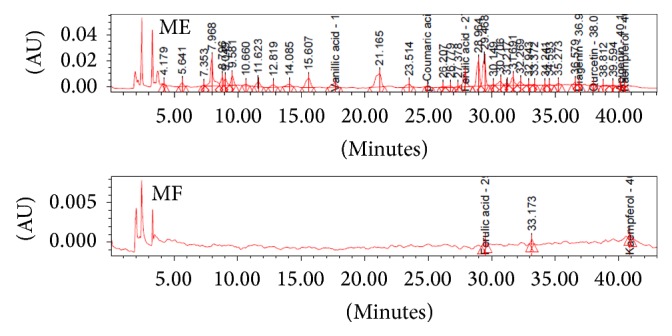
HPLC chromatograms of flavonols detected at 280 nm.

Changes in the composition of organic acids by fermentation process are important factors determining the quality of the final fermentation product. Organic acid composition of each extract revealed the following results. Nine peaks of ME and MF, citric (tR 11.1 minutes), tartaric (tR 11.7 minutes), malic (tR 12.8 minutes), quinic (tR 13.2 minutes), succinic (tR 15.1 minutes), lactic (tR 16.4 minutes), acetic (tR 18.8 minutes), propionic (tR 22.2 minutes), and butyric (tR 27.3 minutes), were identified by HPLC analysis and comparison with standard compounds. Citric (tR 11.1 minutes), malic (tR 12.8 minutes), succinic (tR 15.1 minutes), lactic (tR 16.4 minutes), acetic (tR 18.8 minutes), propionic (tR 22.2 minutes), and butyric (tR 27.3 minutes) were not detected in MF (fermented ME) due to the fact that chemical constituents of ME extracts from* Rhizopus oligosporus* were changed by fermentation process or used as a nutrient for* Rhizopus oligosporus* growth (Figure 1). As fermentation progresses, citric acid is converted into lactic acid and acetic acid, and various organic acids are produced. Nevertheless, it seems that the MF extracts did not cause abnormal fermentation due to high organic acid content at the initial stage of fermentation. This kind of tendency was similar to flavonols composition. Because of analysis of flavonols composition of each extract with 10 flavonols standards, only ferulic acid and kaempferol components were detected in MF fermented extracts (Figure 2). The results suggest that flavonols are degraded to free form or converted to other glycosides due to microbial enzymes in the fermentation process. Respective concentrations of flavonols in ME extracts were 10.05 ppm ± 0.54 ppm for vanillic acid, 0.52 ppm ± 0.13 ppm for p-coumaric acid, 13.16 ppm ± 0.64 ppm for ferulic acid, 0.81 ppm ± 0.09 ppm for diagenin, 4.66 ppm ± 1.9 ppm for quercetin, 2.34 ppm ± 1.7 ppm for apigenin, and 1.55 ppm ± 0.13 ppm for kaempferol (Table 3). Change in flavonols concentration is due to the microbial fermentation process. The total phenolic levels were detected in* Moringa* with ME extracts (0.172 mg/mL), followed by MF extracts (0.038 mg/mL) (Table 4). Generally, polyphenol compounds are one of the widely distributed secondary metabolites in plants. Representative polyphenol compounds include flavonoids, anthocyanin, tannin, catechin, isoflavone, and resveratrol. Therefore, the lower flavonols content of the MF extracts compared to the ME extracts is related to the total phenolic content.

### 3.2. Clinical Skin Severity Score

**Figure 3:**
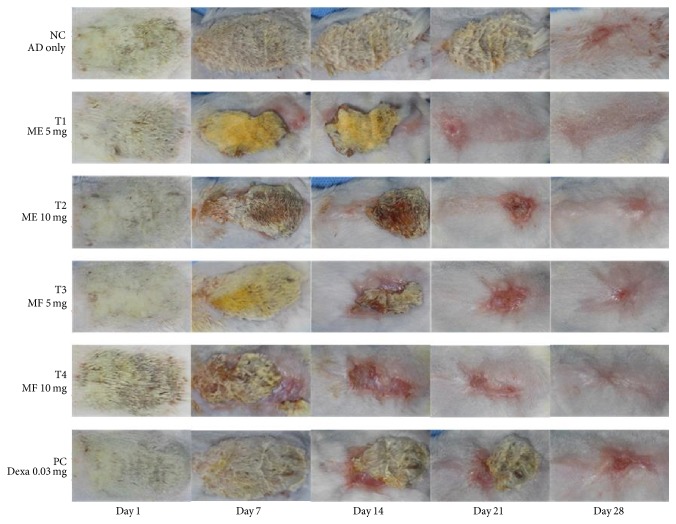
The effect of ME and MF on atopic dermatitis-induced animal model. ME and MF significantly mitigated 5 symptoms: erythema/darkening, edema/population, excoriations, lichenification/prurigo, and dryness at 14 days after treatment.

**Figure 4:**
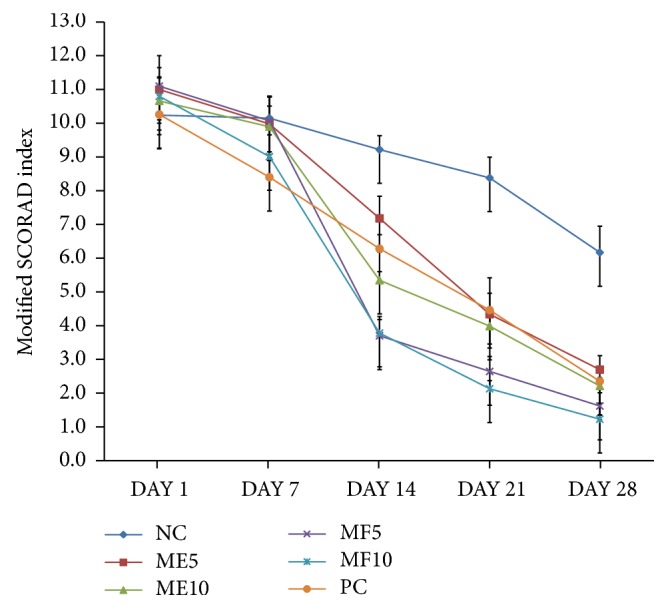
Modified SCORAD index revealed dramatically improved symptoms containing erythema, darkening, edema, papulation, excoriations, lichenification, prurigo, and dryness in ME- and MF-treated groups which are more effective from 14 to 21 days of topical application.

**Figure 5:**
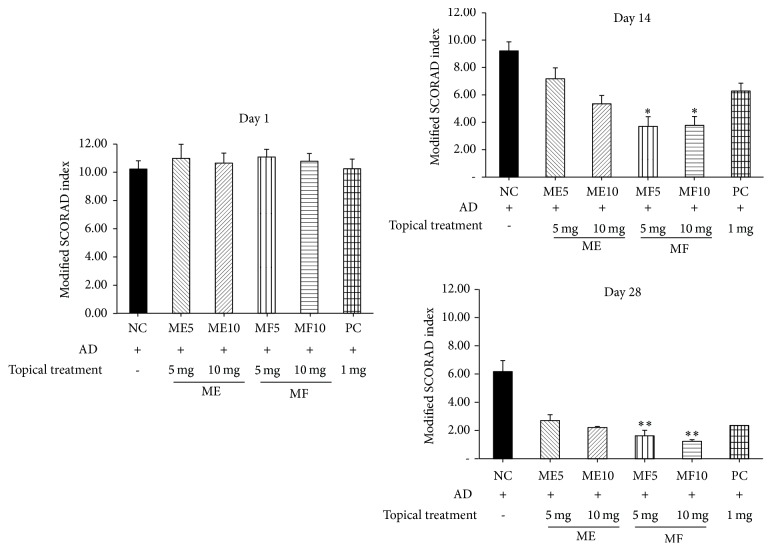
Skin severity score significantly decreased in the MF5 and MF10 groups compared to ME5 and ME10 groups (*p* < 0.05) in 14-week evaluation and (*p* < 0.01) in 28-week evaluation.

To determine if ME or MF can relieve local inflammation in the skin sensitized by DNCB, the modified SCORAD index and scratching frequency were recorded on days 1, 7, 14, and 28, respectively (Table 5). Atopic lesions of mice were observed until 28 days after first treatment. From 14 days to 21 days after treatment, ME and MF remarkably improved atopic symptoms compared to control dexamethasone, an anti-inflammatory reagent (Figure 3). Modified SCORAD index revealed dramatically improved symptoms containing erythema, darkening, edema, papulation, excoriations, lichenification, prurigo, and dryness in ME- and MF-treated groups which are more effective from 14 to 21 days of topical application (Figure 4). There are more statistically significant decreases in MF 5 mg/kg group and MF 10 mg/kg group than ME 5 mg/kg group and ME 10 mg/kg group. As revealed in Figure 5, skin severity score significantly decreased in MF5 and MF10 group than ME5 and ME10 group (*p* < 0.05) in 14-week evaluation and *p* < 0.01 in 28-week evaluation.

### 3.3. Scratching Behavior

**Figure 6:**
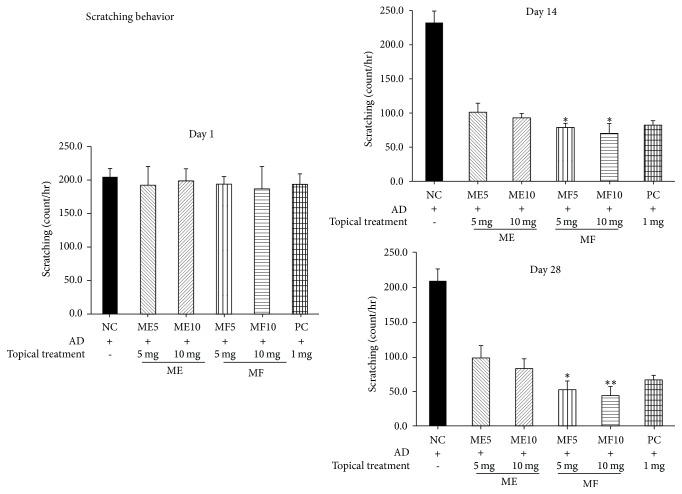
ME reduced the number of scratches and MF exhibited more significantly decreased scratches on day 14 after the start of treatment, accompanied by improvement in eruptions in the modified SCORAD system.

We next investigated effects of treatment of ME and MF on the scratching behavior in mice. As represented in Figure 6, ME reduced the number of scratches and MF exhibited more significantly decreased scratches on day 14 after the start of treatment, accompanied by improvement in eruptions in the modified SCORAD system. On day 28 after treatment, the frequencies of scratching in MF treatment group were significantly lower than control group. Scratching behavior was diminished by treatment of MF (*p* < 0.05 in 5 mg MF treated group and *p* < 0.01 in 10 mg MF treated group, resp., compared with ME treated group). These anti-AD effects were observed in dose-dependent manner with statistical significance.

### 3.4. Serum IgE

**Figure 7:**
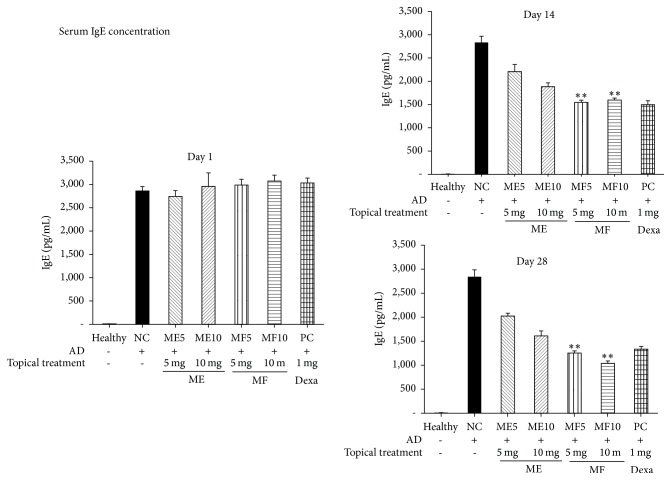
Plasma IgE concentration was highly increased than control group, and increased IgE levels in the AD mice were reduced with MF (*p* < 0.01 in 50 mg/kg and 10 mg/kg, resp., compared with ME-treated group) from day 14 after treatment.

Because AD is a type I IgE-mediated hypersensitivity reaction contributing to immune dysregulation and its major characteristic is hyperproduction of IgE, we examined plasma IgE levels in mice treated with ME or MF and compared them with those of control groups and healthy mice.  Figure 7 reveals that when the mice were induced to the AD, plasma IgE concentration highly increased compared to the control group, and increased IgE levels in the AD mice were reduced with MF (*p* < 0.01 in 50 mg/kg and 10 mg/kg, resp., compared with ME-treated group) from day 14 after treatment. Consistent with skin severity and starching behavior results, MF exhibited much stronger reducing effect on IgE secretion in the plasma of AD mice model compared to ME-treated group; even the ME group revealed anti-AD effect also.

### 3.5. Histopathological Evaluation

**Figure 8:**
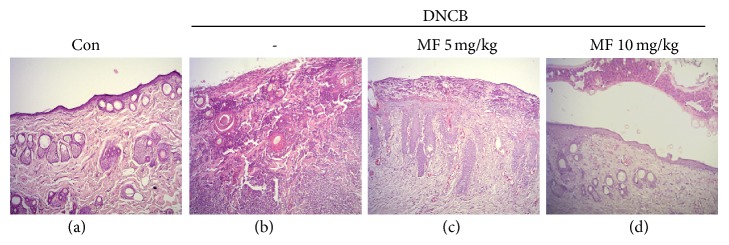
Topical application of MF improved AD-like lesions in a dose-dependent manner according to the histopathological evaluation results.

In Figure 8, the structures of the epidermis, dermis, and skin appendages were well maintained in the skin of untreated control mice (a). DNCB-induced AD-like lesions in mice were characterized by destruction of entire epidermis, marked infiltration of immune cells largely composed of neutrophils, and severe necrotic debris (b). Topical application of MF improved AD-like lesions (c and d). In mice treated with 5 mg/kg of MF, each layer of epidermis was well regenerated and inflammatory cells were rarely observed in the dermis, although necrotic tissue debris was observed on the surface of the epidermis (c). Necrotic debris absolutely desquamated from the epidermis and the skin tissues were recovered to almost normal in mice with treatment of MF 10 mg/kg (d).

## 4. Discussion

We investigated effects of ME and its fermented product using* Rhizopus oligosporus*, MF,* in vitro* and* in vivo* in an AD-like animal model. The results demonstrated that MF effectively reduced clinical features and the index of AD-like animal model's serum IgE level. Considering that AD is the most common skin disease, it is worth noting that clinical recovery from AD-like skin was more clearly observed based on macrography, scratching count, and severity scores in the treatment group. Hyperpigmentation and erythematous lesion eruptions gradually improved significantly in the treatment group in a dose-dependent manner. Mice treated with ME or MF did not reveal clinical signs in the skin after 28 days of topical application compared to dexamethasone-treated group (positive control group). The no-treatment group suffered continuously from skin eruptions and skin injuries in response to scratching behavior after all posttreatment days. ME and MF significantly inhibited the AD clinical signs in a dose-dependent manner with statistical significance. Histopathological evaluations support efficacy of MF that the necrotic debris absolutely desquamated from the epidermis and skin tissues recovered to almost normal in mice with treatment of MF10 group compared to other experimental groups.

Studies investigating genomic and histologic profiling of AD skin lesions have increased recent understanding of disease pathogenesis. Previous studies indicated that* M. oleifera* extract exhibited therapeutic effects during acute and chronic stages of AD [6]. Tsala et al. [14] reported that* M. oleifera* extract could inhibit the early phase of inflammation and play a significant role in chronic inflammation. In addition,* M. oleifera *seeds revealed the same anti-inflammatory effect on acetic acid induced acute colitis in rats in a study by Minaiyan et al. [15]. The mechanism of these effects by* M. oleifera* and their bioconversion product in this study is supposed to have interesting pharmacological efficacies. Further study is needed to determine pharmacokinetics of effective molecules in* M. oleifera* and efficacy mechanism from preliminary study results. Pharmacodynamics and action mechanisms of ME and MF are currently being investigated* in vitro* with a focus on regulatory T cell mechanism, inflammasome participation, Th1/Th2 cytokines, and other related chemokines.

*M. oleifera* belongs to Moringaceae and is 5–10 m high, grown in Asia, Africa, and Arabian countries. It is rich in protein and vitamins, has a high nutritional value, and medically has various pharmacological actions such as hyperglycemia and anti-inflammation and anticancer activities [16].* Moringa* flowers, roots, seeds, leaves, and fruits are rich in phytochemicals such as vitamins, flavonoids, and amino acids. Among the various parts of* Moringa*, leaves are rich in *β*-carotene, protein, vitamin C, and calcium and are used as antioxidants and have been reported to be effective in skin inflammation and skin wound healing [17]. Additionally, extract of* Moringa* contains physiologically active substances such as flavonoids, isothiocyanates, glucosinolates, and thiocarbamates, hepatoprotective effect, and cell suicide and proliferation inhibitory effect of cancer cells [18]. Superiority of Lingua leaves has been proven in several articles [19]. HPLC analysis of ME and MF strongly implies that new constituents or increased constituents may be involved in the antioxidant substance in this preliminary study. Comparing organic acids and flavonols of ME and MF extracts, MF extracts revealed significantly less organic acids and flavonols than ME extracts. It is perceived that microorganisms in the fermentation process used various ingredients in the* Moringa* extract as nutrients or decomposed into other substances. However, further research should be conducted to test these hypotheses. Therefore, we are currently separating compounds from MF extract and identifying new substances using the ^1^H and ^13^C NMR spectra as further study.

In summary, pharmacological profiles of ME and MF were as follows: reduction of clinical features and IgE production suggest that ME and MF are effective for the treatment of AD in a dose-dependent manner. The topical application route may be useful for clinical improvement of AD, even though oral administration should be tested for further study. Bioconversion by fermentation significantly enhances the anti-inflammatory effect of* M. oleifera*. Further studies are necessary to clarify the mechanisms of* M. oleifera* and* M. oleifera* bioconversion product for treatment of AD and relative composition changes or absorbance advantages. Our findings suggest that* M. oleifera* and its bioconversion by* Rhizopus oligosporus*, which has stronger efficacy, could be applicable to develop a potent therapeutic reagent for treatment of AD.

## Figures and Tables

**Table 1:** Operating condition for HPLC.

Instrument	Condition
Model	Prominence (Shimadzu Co.)
Column	Hi-Plex H (7.7 mm × 300 mm I.D.)
Mobile phase	50 mM H_2_SO_4_ (dissolved in water)
Flow rate	20 *μ*L
Oven temperature	65°C
Injection time	30 min

**Table 2:** Mobile phase condition of chromatographic separation (280 nm).

Time	Distilled water (%)	Acetonitrile (%)	Flow (mL/min)
0.00	90.00	10.00	1.00
5.00	90.00	30.00	1.00
30.00	70.00	35.00	1.00
40.00	65.00	10.00	1.00
45.00	90.00	10.00	1.00
50.00	90.00	10.00	1.00

**Table 3:** The flavonols concentrations of ME and MF extracts.

Flavonols	ME	MF
Gallic acid	ND^(1)^	ND
Chlorogenic acid	ND	ND
Vanillic acid	10.05 ± 0.54	ND
Caffeic acid	ND	ND
*Ρ*-Coumaric acid	0.52 ± 0.13	ND
Ferulic acid	13.16 ± 0.64	0.94 ± 0.85
Diagenin	0.81 ± 0.09	ND
Quercetin	4.66 ± 1.9	ND
Apigenin	2.34 ± 1.7	ND
Kaempferol	1.55 ± 0.13	2.05 ± 0.25

Mean ± SD. ^(1)^ND: not dectected.

**Table 4:** The contents of total polyphenol from ME and MF extracts.

Samples	Contents (mg/mL)
ME	0.172 ± 0.002
MF	0.038 ± 0.010

Mean ± SD.

**Table 5:** Modified SCORAD index.

	Day 1	Day 7	Day 14	Day 21	Day 28
NC	10.2 ± 0.6	10.2 ± 0.7	9.2 ± 0.4	8.4 ± 0.6	6.2 ± 0.8
ME5	11.0 ± 1.0	10.0 ± 0.8	7.2 ± 0.7	4.3 ± 1.1	2.7 ± 0.4
ME10	10.7 ± 0.7	9.9 ± 0.6	5.3 ± 0.3	4.0 ± 0.3	2.2 ± 0.1
MF5	11.1 ± 0.5	10.1 ± 0.7	3.7 ± 0.5	2.6 ± 0.4	1.6 ± 0.4
MF10	10.8 ± 0.5	9.0 ± 0.6	3.8 ± 0.5	2.1 ± 0.2	1.2 ± 0.1
PC	10.3 ± 0.7	8.4 ± 0.6	6.3 ± 0.4	4.5 ± 0.5	2.4 ± 0.1

## References

[1] Chen M., Ding P., Yang L. (2017). Evaluation of Anti-Inflammatory Activities of Qingre-Qushi Recipe (QRQS) against Atopic Dermatitis: Potential Mechanism of Inhibition of IL-33/ST2 Signal Transduction. *Evidence-Based Complementary and Alternative Medicine*.

[2] Spergel J. M., Paller A. S. (2003). Atopic dermatitis and the atopic march. *The Journal of Allergy and Clinical Immunology*.

[3] Choopani R., Mehrbani M., Fekri A., Mehrabani M. (2017). Treatment of Atopic Dermatitis From the Perspective of Traditional Persian Medicine: Presentation of a Novel Therapeutic Approach. *Evidence-Based Complementary and Alternative Medicine*.

[4] Kobayashi H., Ishii M., Takeuchi S. (2010). Efficacy and safety of a traditional herbal medicine, hochu-ekki-to in the long-term management of Kikyo (Delicate Constitution) patients with atopic dermatitis: a 6-month, multicenter, double-blind, randomized, placebo-controlled study. *Evidence-Based Complementary and Alternative Medicine*.

[5] Leone A., Spada A., Battezzati A., Schiraldi A., Aristil J., Bertoli S. (2015). Cultivation, genetic, ethnopharmacology, phytochemistry and pharmacology of Moringa oleifera leaves: An overview. *International Journal of Molecular Sciences*.

[6] Choi E.-J., Debnath T., Tang Y., Ryu Y.-B., Moon S.-H., Kim E.-K. (2016). Topical application of Moringa oleifera leaf extract ameliorates experimentally induced atopic dermatitis by the regulation of Th1/Th2/Th17 balance. *Biomedicine & Pharmacotherapy*.

[7] Fard M. T., Arulselvan P., Karthivashan G., Adam S. K., Fakurazi S. (2015). Bioactive extract from Moringa oleifera inhibits the pro-inflammatory mediators in Lipopolysaccharide stimulated macrophages. *Pharmacognosy Magazine*.

[8] Zaffer M., Ahmad S., Sharma R., Mahajan S., Gupta A., Agnihotri R. K. (2014). Antibacterial activity of bark extracts of Moringa oleifera Lam. against some selected bacteria. *Pakistan Journal of Pharmaceutical Sciences*.

[9] Vongsak B., Mangmool S., Gritsanapan W. (2015). Antioxidant activity and induction of mRNA expressions of antioxidant enzymes in HEK-293 cells of *Moringa oleifera* leaf extract. *Planta Medica*.

[10] Chung T. H., Kang T. J., Cho W.-K. (2012). Effectiveness of the novel herbal medicine, KIOM-MA, and its bioconversion product, KIOM-MA128, on the treatment of atopic dermatitis. *Evidence-Based Complementary and Alternative Medicine*.

[11] Feng Y., Zhang M., Mujundar A., Gao Z. (2017). Recent Research Process of Fermented Plant Extract: a Review. *Trends in Food Science & Technology*.

[12] Ab Jalil A., Abdullah N., Alimon A. R., Abd-Aziz S. (2014). Nutrient Enhancement of Ground Sago (Metroxylon sagu Rottboll) Pith by Solid State Fermentation with Rhizopus oligosporus for Poultry Feed. *Journal of Food Research (JFR)*.

[13] Dorman H. J. D., Peltoketo A., Hiltunen R., Tikkanen M. J. (2003). Characterisation of the antioxidant properties of de-odourised aqueous extracts from selected *Lamiaceae* herbs. *Food Chemistry*.

[14] Tsala D. E., Simplice F. H., Thierry B. N. M., Justin B., Emmanuel N. (2013). Anti-inflammatory activity of hot water extract of moringa oleifera lam in rats. *International Journal of Diary Technology*.

[15] Minaiyan M., Asghari G., Taheri D., Saeidi M., Nasr-Esfahani S. (2014). Anti-inflammatory effect of Moringa oleifera Lam. seeds on acetic acid-induced acute colitis in rats. *Avicenna journal of phytomedicine*.

[16] Jung I. L. (2014). Soluble extract from *Moringa oleifera* leaves with a new anticancer activity. *PLoS ONE*.

[17] Lako J., Trenerry V. C., Wahlqvist M., Wattanapenpaiboon N., Sotheeswaran S., Premier R. (2007). Phytochemical flavonols, carotenoids and the antioxidant properties of a wide selection of Fijian fruit, vegetables and other readily available foods. *Food Chemistry*.

[18] Sreelatha S., Jeyachitra A., Padma P. R. (2011). Antiproliferation and induction of apoptosis by *Moringa oleifera* leaf extract on human cancer cells. *Food and Chemical Toxicology*.

[19] de Mesquita M. L., Grellier P., Mambu L., de Paula J. E., Espindola L. S. (2007). In vitro antiplasmodial activity of Brazilian Cerrado plants used as traditional remedies. *Journal of Ethnopharmacology*.

